# 

**Published:** 1891-08

**Authors:** 


Vol. XII.] PUBLISHED MONTHLY AT THREE DOLLARS A YEAR. SINGLE COPIES, THIRTY CENTS. [No. 8.

Entered at the Post Office, February 3rd, 1891, at New York as Second-class Matter.
Copyright, 1891, by W. A. Conklin, and R. S. Huidekoper.

### THE JOURNAL OF COMPARATIVE MEDICINE AND VETERINARY ARCHIVES.

EDITED BY
W. A. CONKLIN, Ph. D., D. V. S.,
*Director of Zoological Gardens, New York.*
RUSH SHIPPEN HUIDEKOPER, M. D., Veterinarian, (*Alfort.*)
*Professor of Sanitary Medicine and Veterinary Jurisprudence, American Veterinary College, New York.*

AUGUST, 1891.

All communications and payments should be addressed to

MANAGING EDITOR JOURNAL COMP. MED. AND VET. ARCH.

Forty-second Street and Park Avenue, New York.

Printed for the Editors by

WILLIAM R. JENKINS, Publisher,

New York.

LONDON: BALLIERE, TINDALL & COX.

